# Lysine Carboxymethyl Cysteinate (LCC) Protects the Epidermis from UVB-Induced Barrier Damage Through the Activation of Autophagy

**DOI:** 10.3390/biology15080601

**Published:** 2026-04-10

**Authors:** Xue Xiao, Hong Zhang, Xuelan Gu

**Affiliations:** Unilever R&D Shanghai, No. 66 Linxin Road, Shanghai 200335, China

**Keywords:** lysine carboxymethyl cysteinate, glutathione precursor, autophagy, barrier protection, UVB protection

## Abstract

Lysine carboxymethyl cysteinate (LCC) has been identified as a glutathione (GSH) precursor for the use of cosmetic products, providing a defense against oxidative stress by elevating GSH levels, and mitigating UVB-induced pigmentation and barrier disruption. In this study, the protective efficacy of LCC on epidermal barrier integrity under UVB irradiation was systematically evaluated and its underlying mechanisms were investigated. Results from the UVB-exposed 3D living skin equivalent model (LSE) indicated that LCC effectively restored UVB-induced reductions in epidermal living cell thickness by 9.67%. In addition, LCC markedly increased the expression of key biomarkers related to cornified envelope (CE) formation and skin hydration, including transglutaminase 1, involucrin, loricrin and aquaporin 3 by 104.80%, 121.67%, 218.63% and 388.39%, respectively, compared with the UVB group. Transcriptomics analysis in human primary keratinocytes further revealed that LCC regulated multiple biological functions, including glutathione synthesis pathway, oxidation response, inflammatory process, and notably autophagy. After confirming LCC’s potential in boosting autophagy-associated gene expression (*p*-value < 0.05) and autophagy activity (*p*-value < 0.01) in keratinocytes, functional validation in the same model confirmed that LCC counteracted UVB-induced suppression of genes involved in barrier formation, particularly those associated with CE development and autophagy, while these protective effects were abolished by chloroquine, an autophagy inhibitor. Findings from the UVB-exposed LSE model further substantiated this mechanism. Collectively, these results demonstrate that LCC safeguards the epidermis from UVB-induced cornification abnormalities through the activation of autophagy.

## Simple Summary

Lysine carboxymethyl cysteinate (LCC) is an ingredient that helps the skin generate more glutathione, a natural antioxidant responsible for defending cells against environmental aggressors and pathogens. Studies have shown that LCC exerts protective benefits against UVB-induced pigmentation and disruptions in barrier function. Given the significant role of autophagy in skin cells’ response to UV exposure, in this study, we investigated the protective mechanisms of LCC against UVB damage using human skin cells and a laboratory-grown 3D skin model. The data indicates that LCC significantly activates autophagy activity and restores essential barrier proteins against UVB exposure, while these protective effects are abolished by the presence of an autophagy inhibitor. Overall, our results suggest that LCC contributes to the preservation of skin barrier integrity during UVB exposure, at least partially through activation of autophagy.

## 1. Introduction

Skin, the largest organ of the human body, serves as the primary interface between the internal milieu and the external environment. Structurally, human skin consists of two major layers, epidermis and dermis, with the epidermis layer forming the essential barrier that defends against environmental insults, like pathogens, toxic agents, and ultraviolet (UV) radiation. Among these stressors, UV irradiation represents the most prevalent and impactful environmental exposure. UV exposure can affect skin structure and functions, leading to clinical manifestations including sunburn, hyperpigmentation and photoaging. In epidermis, UVB is the most biologically harmful component of the UV spectrum, disrupting the skin barrier through inducing DNA damage and keratinocyte apoptosis, inflammatory activation [1] and excessive oxidative stress by stimulating reactive oxygen species (ROS) production [2]. Elevated ROS influence multiple cellular process, including causing DNA damage by oxidizing nucleotide bases and generating DNA strain breaks [3], dysregulating transcription factors including nuclear factor-kappa B (NF-κB), activator protein-1 (AP-1) and nuclear factor erythroid 2-related factor 2 (Nrf2), as well as activating immune cell response [4]. Therefore, maintaining redox homeostasis is crucial for preserving skin health, particularly under UV exposure.

To counteract oxidative stress and maintain cellular redox homeostasis, cells rely on an intricate antioxidant network, among which the glutathione (GSH) system plays a central role. Glutathione, a triple-peptide composed of glycine, cysteine, and glutamate, functions as a major antioxidant by directly neutralizing ROS, scavenging free radicals and participating in multiple antioxidant pathways [5]. In skin, glutathione participates in various physiological processes, like aging, pigmentation and notably barrier formation. The *Gclc* knockout study in mice [6] demonstrated that glutathione was indispensable for maintaining cellular redox balance, keratinocyte viability and proper differentiation. Disruption of glutathione synthesis resulted in keratinocyte DNA damage and increased cell death, and dysregulation of keratinization and differentiation.

Given its essential redox-protective functions, glutathione has been considered a promising active ingredient for skin protection. However, the inconsistent skin efficacy of glutathione following topical application has limited its broader utilization in cosmetic formulations. These challenges have led to the development of glutathione precursor strategies. Recently, lysine carboxymethyl cysteinate (LCC) has emerged as a novel glutathione precursor [7]. LCC has been shown to penetrate and enhance de novo glutathione synthesis in epidermis. It was also able to reduce ROS production under blue light exposure, and attenuate UVB-induced pigmentation, barrier function interruption, and inflammatory cytokine release.

Although LCC has emerged as a promising glutathione precursor with demonstrated antioxidant and photoprotective effects, its mechanisms in skin barrier protection remain largely undefined. Therefore, the present study investigates the UVB-protective effect of LCC using a 3D living skin equivalent (LSE) model, elucidating the autophagy-modulating role of LCC in primary normal human epidermal keratinocyte and further examines the involvement of autophagy in LCC-mediated UVB protection in both keratinocyte and LSE models.

## 2. Materials and Methods

### 2.1. Human Primary Epidermal Keratinocyte Treatment

Primary normal human epidermal keratinocyte (NHEK) was maintained at 37 °C with 5% CO_2_. When they reached 80% confluence, the cells were seeded into a 12-well culture plate. After 48 h, the cells were treated with LCC (Sinerga, Cat: HairApp, LCC purity ≥ 98.00%, Gorla Maggiore, Italy) at a concentration of 100 μM for 24 h. Then, cells were lysed with 500 µL TRIzol medium and stored at −80 °C for RNAseq.

NHEK used for qPCR validation was purchased from Guangdong BioCell Biotechnology Co., Ltd. (Lot: Ep24070801, Guangzhou, China). The keratinocytes were seeded in 6-well plates and cultured before treatment. When the cell confluency reached 40–60%, LCC at a final concentration of 100 μM was added to the cell culture medium, and the cells were incubated for another 24 h before harvest. In the control group, the culture medium was refreshed.

NHEK used for autophagy activity measurement was purchased from Promocell (Lot: 425Z026.2, Heidelberg, Germany). The cells were seeded on 96-well plates with black walls and cultured for 24 h before treatment. The next day, the culture medium with 100 μM LCC was added to the cell. After incubation for another 24 h, the cells were used for autophagy activity analysis.

### 2.2. UVB Exposed Keratinocyte Treatment

NHEKs used for autophagy activity measurement (Promocell, Lot: 0012602.2, Heidelberg, Germany) were seeded in a 96-well plate and incubated for 24 h. On the second day, the cells of the UV group and the treatment groups were exposed to 200 mJ/cm^2^ UVB. Then the cells in treatment groups were further cultured in medium containing 100 μM LCC. After incubation for 24 h, the cells were used for autophagy activity analysis.

NHEKs used for qPCR analysis were seeded in a 12-well plate and incubated for 24 h. On the second day, the cells of the UV group and the treatment groups were exposed to 200 mJ/cm^2^ UVB. Then the cells in treatment groups were further cultured in medium containing 100 μM LCC with or without 2 μM chloroquine (CQ). After incubation for 24 h, the cells were collected for qPCR.

### 2.3. UVB Exposed 3D Skin Equivalent Model Treatment

LSE model (Epikuitis^®^, Lot: ES250102 and ES251004, BioCell Biotechnology Co., Ltd., Guangzhou, China) was used to study the UVB protection effects of LCC. To investigate the protective function of LCC. The LSE model in the UVB and treatment groups were exposed to 200 mJ/cm^2^ UVB daily from Day 1 to Day 3. The models in the treatment group were topically treated with 5 μL 0.1% LCC before UVB exposure, and the sample was spread evenly on the surface of the model with a soft silicone stick. After the last treatment, all LSE models were incubated for 24 h before being collected for further analysis.

In the study to discover the role of autophagy and LCC in UVB protection, the LSE was treated with UVB in the same procedure as described previously. In the treatment group, 100 μM LCC, and its combination with 2μM CQ, were applied on the surface of the LSE model before each UVB exposure from Day 1 to Day 3. The models were collected 24 h after the last treatment.

### 2.4. Transcriptomic Study

RNA sequencing was conducted using Illumina NovasSeq 60000 (Illumina, San Diego, CA, USA). Clean reads were mapped to the human genome (GRCh38). DESeq2 (version 1.30.1) was used for differential analysis, and differential expression genes were identified using a cut-off of *p*-value < 0.05 and log2 (fold change) > 1. Biological functions involved by differentially expressed genes were analysed using clusterProfler (version 4.12.6), pathway analysis was conducted using Ingenuity Pathway Analysis (IPA, Qiagen, Hilden, Germany). Gene Set Enrichment Analysis was conducted using fgsea (version 1.30.0).

### 2.5. RNA Extraction and qPCR

After LCC treatment, the keratinocytes were rinsed with PBS twice, and AG RNAex Pro RNA (Accurate Biotechnology, AG21102, Changsha, China) was added to lyse the cells. Then, chloroform was added to each well to extract RNA. The total RNA was measured using a Nanodrop spectrometer (Thermo Fisher Scientific, Rockford, MA, USA) and reverse transcribed to generate cDNA using the Evo M-MLV RT Premix for qPCR (Accurate Biotechnology, Cat: AG11728, Changsha, China) according to the manufacturer’s protocol. The expression levels of *LC3* (F:5′-GAACTGAGCTGCCTCTACCG-3′; R:5′-GGGACAACCCTAACACGACC-3′) were analyzed using a SYBR Green Pro Taq HS Premix Pro Taq HS qPCR Kit (Accurate Biotechnology, AG11701, Changsha, China). The actin beta (*ACTB*) gene was used as the housekeeping gene. The expression levels of the target gene were calculated by 2^−ΔΔCT^.

Total RNA collected from UVB-exposed keratinocytes was extracted with RNAiso Plus (Takara Biomedical Technology, Cat: 9108, Beijing, China). The concentration of RNA was measured by the Nanodrop spectrometer (Thermo Fisher Scientific, Waltham, MA, USA), and then the RNA was reverse transcribed to cDNA with PrimeScript RT Master Mix (Takara Biomedical Technology, Cat: RR036, Beijing, China). qPCR was performed with SYBR^®^ Premix Ex Taq™ (Takara Biomedical Technology, Cat: RR420A, Beijing, China) on the ABI Vii 7 Real-Time PCR System (Applied Biosystems, Thermo fisher scientific, Carlsbad, CA, USA) with primers of *LC3B* (F:5′-GAGAAGCAGCTTCCTGTTCTGG-3′; R:5′-GTGTCCGTTCACCAACAGGAAG-3′), *AQP3* (F:5′-CCGTGACCTTTGCCATGTGCTT-3′; R:5′-TTGTCGGCGAAGTGCCAGATTG-3′), *TGM1* (F:5′-GAACGACTGCTGGATGAAGAGG-3′; R:5′-CTTGATGGACTCCACAGAGCAG-3′), *LOR* (F:5′-GTCTGCGGAGGTGGTTCCTCT-3′; R:5′-TGCTGGGTCTGGTGGCAGATC-3′) and *IVL* (F:5′- GGTCCAAGACATTCAACCAGCC-3′; R:5′-TCTGGACACTGCGGGTGGTTAT-3′). *GAPDH* was selected as the housekeeping gene. The calculation was the same as previously described.

### 2.6. Autophagy Activity Measurement

After treatment, the culture medium was removed, and the NHEKs were washed with PBS three times. Then the NHEKs were incubated with CYTO-ID^®^ Autophagy detection kit (Enzo Biochem, Cat: ENZ-51031, Long Island, NY, USA) according to the manufacturer’s instructions. The fluorescence was measured with Spark^®^ Microplate Reader (Tecan, Männedorf, Switzerland). The total autophagy activity was normalized to Hoechst fluorescent intensity.

### 2.7. H&E, Immunohistochemistry, and Immunofluorescence Staining

After treatment, the harvested samples were fixed in 4% paraformaldehyde solution (Biosharp, Cat: BL539A, Beijing, China). Then the samples were dehydrated and embedded in paraffin. After fixation, the tissue was sectioned into 5 μM and stored for H&E staining and immunochemistry staining.

The prepared tissue sections were deparaffinized and rehydrated through a graded ethanol series and stained with hematoxylin and eosin (H&E) (Beyotime Biotechnology, Cat: C0107 and C0109, Shanghai, China) for histological evaluation. To analyze the thickness of the living cell layer in the tissue, the border of the living cell in the epidermal layer was drawn, and the average distance between the border lines was calculated with Image Pro Plus (version 6.0, Media Cybernetics Inc., Rockville, MD, USA).

Sections from EpiKutis^®^ reserved for immunohistochemistry staining were analyzed for loricrin and aquaporin 3, and were prepared with the primary antibodies against loricrin (Abcam, Cat: ab198994, Waltham, MA, USA) and aquaporin 3 (Abcam, Cat: ab125219, Waltham, MA, USA), and a secondary antibody against the primary antibody. After antibody incubation, the sections were incubated with a DAB chromogen (Maxim Biotechnologies, Cat: DAB-1031, Fuzhou, China) and dehydrated before being sealed.

EpiKutis^®^ sections reserved for immunofluorescence staining were incubated with primary antibody against transglutaminase 1 (Proteintech, Cat: 12912-3-AP, Wuhan, China) and antibody against involucrin (Abcam, Cat: ab181980, Waltham, MA, USA), followed by incubation with secondary antibody against primary antibody. Then the sections were stained with Hochest (Beyotime Biotechnology, Cat: C1022, Shanghai, China) and dehydrated before being sealed.

The images were acquired under microscopes (Olympus, BX53 and BX43, Center Valley, PA, USA), and the protein expression levels were quantified with Image Pro Plus.

### 2.8. Statistics

The results from in vitro models are in triplicate and presented as mean ± standard deviation (SD) in the figures. Statistical significance was calculated with the Student’s T-test with two tails and equal SD (*p* < 0.05 was considered significant).

## 3. Results

### 3.1. LCC Protected 3D Skin Equivalent Model Against UVB-Induced Damage

**Figure 1:**
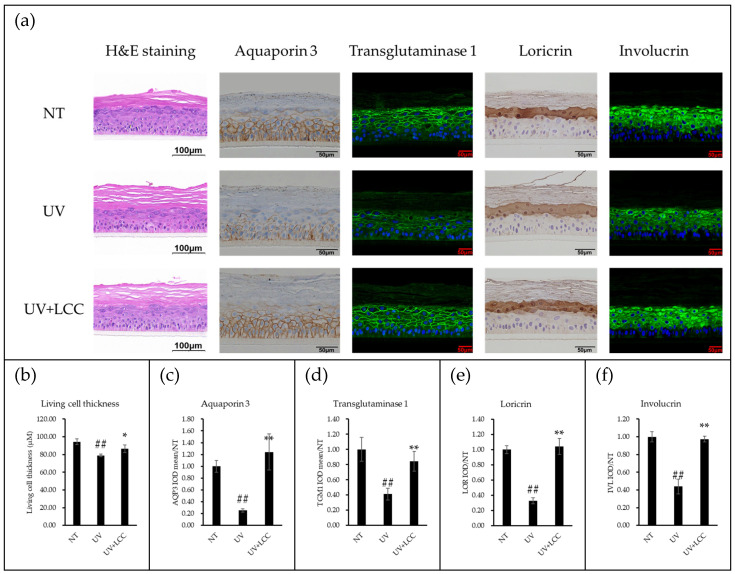
LCC protects the skin barrier against UVB-induced damage in the LSE model. (**a**) H&E staining, immunofluorescence staining, and immunohistochemistry staining of the LSE model; (**b**) shows the analysis of living cell thickness from the H&E staining; (**c**–**f**) shows the quantification of the integrated optical density (IOD) of the image. ## indicates *p*-value < 0.01 compared to NT. * and ** indicate *p*-value less than 0.05 and *p*-value less than 0.01 compared to UVB, respectively. Data are presented as mean ± SD, and *n* = 3.

To evaluate the UV protective efficacy of LCC, the LSE model was exposed to UVB irradiation and subsequently treated topically with LCC. Hematoxylin and Eosin (H&E) staining was conducted for epidermal thickness assessment; the levels of aquaporin 3, transglutaminase 1, loricrin, and involucrin, four biomarkers associated with skin barrier formation and integrity, were assessed using immunostaining. UVB exposure led to a reduction in living cell thickness in the LSE model, accompanied by decreased expression levels of those four biomarkers (Figure 1a). Topical application of LCC effectively mitigated these UVB-induced alterations, restoring both epidermal thickness and biomarker expression levels (Figure 1b–e).

### 3.2. LCC Regulated Autophagy Activities in NHEKs

#### 3.2.1. Transcriptomic Analysis Suggests LCC May Regulate Autophagy

**Figure 2:**
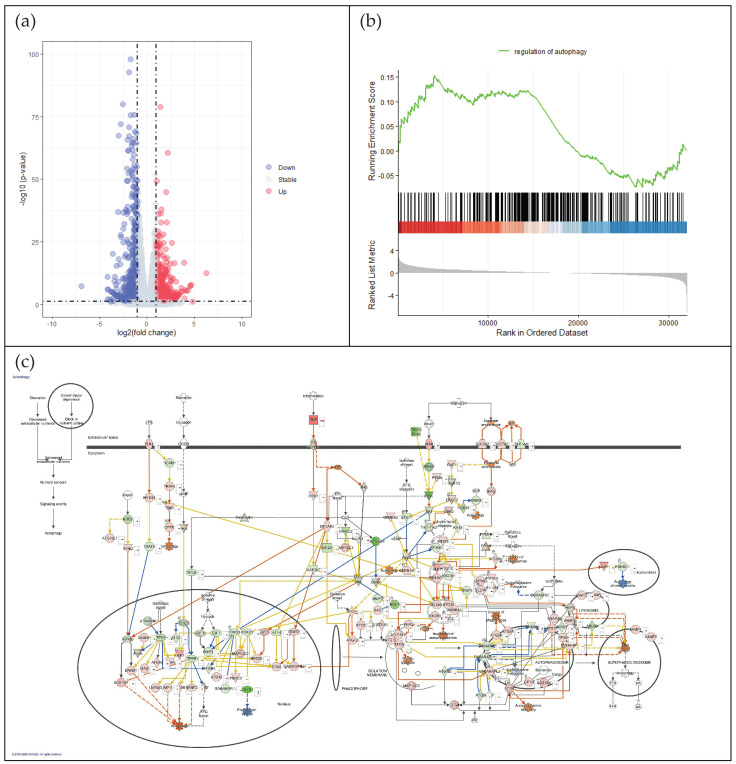
LCC regulates the autophagy pathway in NHEKs. (**a**) shows a volcano plot of gene changes under LCC treatment. (**b**) indicates an autophagy-enriched score by gene expression change under LCC using GSEA analysis. (**c**) demonstrates autophagy pathway activity by overlaying gene expression changes under LCC on the IPA pathway.

To explore mechanistic insights into LCC’s protective effect in skin, a transcriptomics study was conducted. Firstly, the differential expression analysis showed that there were more than 7000 genes that were significantly changed, and about 1600 of them have an absolute fold change bigger than 2 under 100 μM LCC (Figure 2a). The top differentially expressed genes ranked by fold change are listed in Table S1. LCC was demonstrated to act as glutathione to protect skin from oxidative stress [7]. The RNA-seq data also showed that oxidation-related genes like *GCLC*, *GCLM*, *GPX1*, *GPX4*, *CAT*, and *TXNRD1* were significantly changed. Meanwhile, cytokines like *IL1A, IL1B* were downregulated by LCC, and *TGFB1, TIMP1* were upregulated. Interestingly, some autophagy-related genes like *ATG10*, *ATG12*, *ATG5*, *LAMP2*, *SQSTM1*, and *ULK1* were regulated by LCC as an indication of its function on autophagy. GO enrichment analysis and GSEA analysis showed that autophagy-related biological processes, such as regulation of autophagy, macroautophagy, microautophagy, and chaperone-mediated autophagy, were involved in these significantly changed genes (Figure 2b). Overlaying expression of gene on the autophagy pathway in IPA, it clearly showed that the autophagy pathway was predicted to be activated by LCC (Figure 2c).

#### 3.2.2. Effect of LCC on LC3 Expression and Autophagy Activity in NHEKs

**Figure 3:**
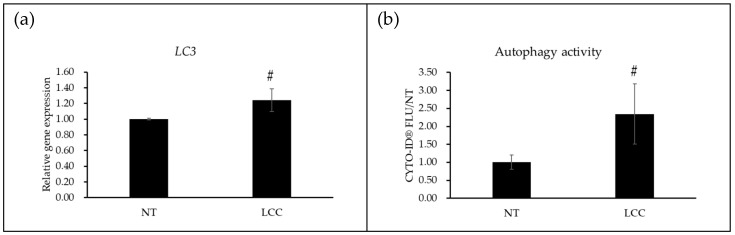
LCC activates autophagy in NHEKs. (**a**) demonstrates the qPCR result in NHKEs. (**b**) shows the autophagy activity in NHKEs. # indicated a *p*-value less than 0.05. Data are presented as mean ± SD, and *n* = 3.

To validate the autophagy regulation function of LCC, both qPCR analysis and an autophagy activity assay were conducted in NHEKs. The qPCR result demonstrated a significant upregulation of *LC3* expression following LCC treatment (Figure 3a). Consistent with gene expression elevation, the result from Cyto-ID which detected the autophagosome and autolysosome further confirmed that LCC markedly enhanced autophagy activity in NHEKs (Figure 3b).

### 3.3. LCC Protected Skin Barrier Through Activating Autophagy Pathways

#### 3.3.1. Barrier Protective Activity of LCC in UVB-Exposed Keratinocytes

**Figure 4:**
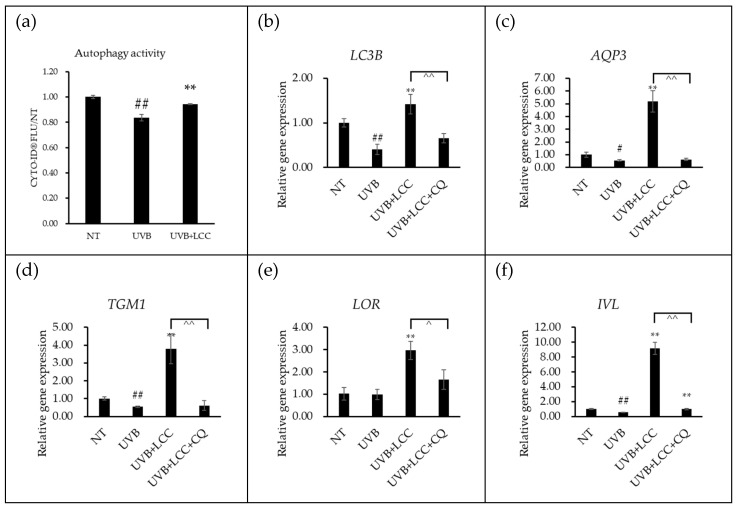
LCC protected skin from UVB-induced barrier damage through autophagy activation in NHEKs. (**a**) showed the autophagy activity in NHEKs. (**b**–**f**) demonstrated the qPCR result in NHKEs. # and ## indicated a *p*-value < 0.05 and *p*-value < 0.01 compared to NT, respectively. ** indicate *p*-value < 0.01 compared to UVB. ^ and ^^ indicated *p*-value < 0.05 and *p*-value < 0.01 of comparison between groups. Data are presented as mean ± SD, and *n* = 3.

To elucidate the involvement of autophagy activity in the protective effects of LCC, NHEKs were employed to examine whether LCC can alleviate UVB-induced barrier impairment through autophagy activation. The Cyto-ID assay showed that LCC restored autophagy activity suppressed by UVB (Figure 4a). Consistently, qPCR results revealed that UVB exposure markedly reduced the expression of a *LC3B*, whereas LCC treatment effectively restored its expression level. However, LCC co-treatment with CQ, an autophagy inhibitor, substantially attenuated the LCC-mediated restoration (Figure 4b). The expression of barrier-associated genes, including *AQP3*, *IVL*, *LOR*, *TGM1*, followed a similar pattern. UVB exposure suppressed expression levels of these genes; the treatment of LCC significantly restored those changes, moreover, LCC’s restoration activity was diminished in NHEKs with presence of CQ (Figure 4c–f), confirming the requirement of autophagic activity for LCC-mediated gene regulation.

#### 3.3.2. LCC Protected Skin Barrier Partially Through Autophagy Activation

**Figure 5:**
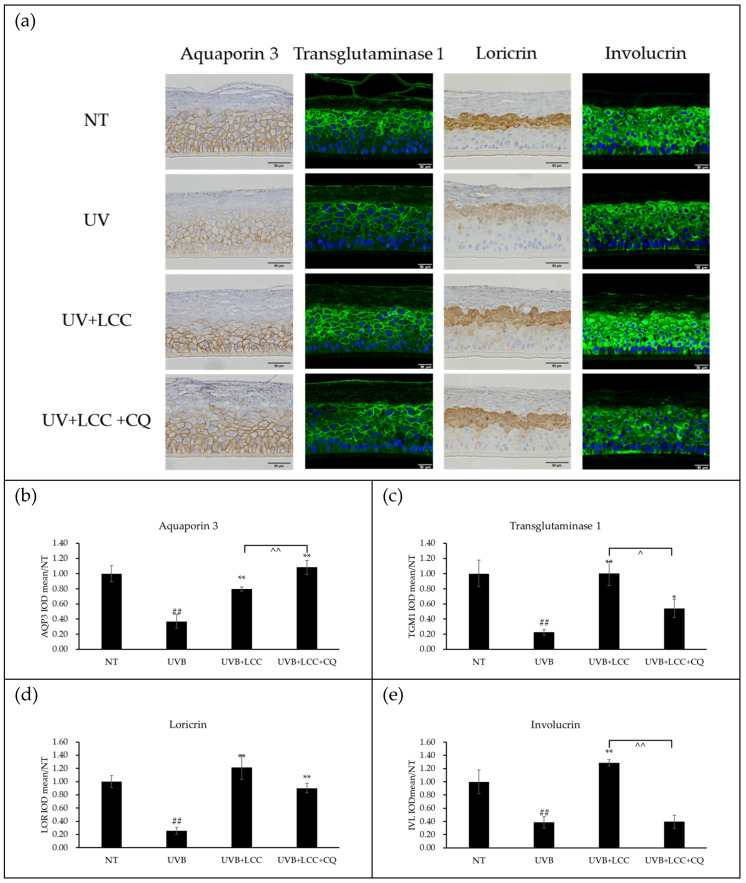
LCC protects skin barrier functions against UVB through autophagy. (**a**) Immunofluorescent and immunohistochemistry staining of UVB-exposed LSE model (scale bar: 50 μm); (**b**–**e**) shows the quantification of IOD of the image as compared with NT. ## indicates *p*-value < 0.01 compared to NT. * and ** indicates *p*-value less than 0.05 and *p*-value less than 0.01 compared to UVB, respectively. ^ and ^^ indicated *p*-value less than 0.05 and *p*-value less than 0.01 of comparison between groups. Data are presented as mean ± SD, and *n* = 3.

To further evaluate the UVB-protective capability of LCC on skin barrier formation and function, LCC was applied to the LSE model in the presence or absence of CQ. LCC treatment restored the expression levels of transglutaminase 1, loricrin, and involucrin; however, these restorative effects were partially or fully abrogated when autophagy was inhibited (Figure 5). Notably, CQ markedly compromised LCC-mediated recovery of transglutaminase 1 and involucrin, while having minimal impact on LCC-induced restoration of loricrin (Figure 5c–e). Interestingly, co-treatment with CQ further enhanced aquaporin 3 expression beyond the level achieved by LCC alone (Figure 5b).

## 4. Discussion

The epidermis serves as the primary protective barrier against assaults from the environment, and its integrity depends on the tight regulation of the balance between keratinocyte proliferation and differentiation. Disruption of this balance is closely associated with various skin disorders including atopic dermatitis, psoriasis, ichthyosis vulgaris, and epidermal malignancies [8]. UVB irradiation is a well-recognized extrinsic factor that perturbs epidermal homeostasis by inducing DNA lesions, impairing DNA repair, and ultimately causing mutations in genes essential for proliferation and differentiation [9,10]. Previous studies indicated that UVB exposure resulted in marked morphological and functional alterations such as thickened stratum corneum, disrupted lipid intercellular structures, interrupted cell cohesion and mechanical integrity, and increased trans-epidermal water loss [11,12,13]. Glutathione plays a central role in mitigating UVB-induced oxidative damage [14,15]. As a newly identified glutathione precursor, LCC demonstrates efficient epidermal penetration and supports *de novo* glutathione synthesis. In agreement with its antioxidant properties, LCC significantly attenuated UVB-induced ROS accumulation and rescued the decline of key barrier proteins, such as filaggrin. In this study, the barrier-protective functions of LCC were comprehensively investigated, and novel mechanisms beyond its conventional antioxidant activity were elucidated.

In the UVB-irradiated 3D living skin equivalent (LSE) model, LCC restored epidermal thickness, reflecting its beneficial impact on both keratinocyte proliferation and differentiation. Subsequently, biomarkers associated with distinct aspects of epidermal barrier function were investigated. Aquaporin 3, a well-known water and glycerol channel located on the plasma membrane, is critical for keratinocyte proliferation and differentiation [16]. Loricrin and involucrin are essential components of cornified envelopes that maintain the “bricks and mortar” architecture of epidermis. Transglutaminase 1, a plasma membrane protein enriched in the granular and spinous layer, plays an important role in skin barrier formation by facilitating the crosslinking of cornified envelope proteins, including filaggrin, loricrin, involucrin, and keratin [17,18,19]. Our results demonstrated that LCC effectively restored UVB-induced reduction of aquaporin 3, transglutaminase 1, loricrin, and involucrin expression (Figure 1). These findings underscore the barrier protection function of LCC, driven in part by its glutathione boosting capability, while suggesting that other underlying mechanisms merit further investigation.

To elucidate the molecular mechanisms underlying these protective effects, RNA-seq analysis was conducted on LCC-treated keratinocytes. LCC modulated several key pathways, particularly enhancing glutathione metabolism and downregulating inflammatory signaling. Notably, genes associated with autophagosome and autolysosome were significantly upregulated (Figure 2c). Follow-up qPCR for *LC3*, which codes the essential protein for autophagosome formation-microtubule-associated protein 1 light chain 3, together with Cyto-ID assays detecting autophagosome and autolysosome, confirmed that LCC activated autophagy by promoting autophagosome formation (Figure 3). Autophagy is a cellular process conserved in eukaryotes, characterized by degrading and recycling cellular organelles, certain proteins, and damaged DNA with lysosomes. It plays an important role in maintaining cellular homeostasis and adapting to starvation and environmental stress, and it shows a pleiotropic role in skin [20]. Autophagy is increasingly recognized as a critical cellular process in skin, influencing skin aging [21], pigmentation [22] and skin barrier functions [23]. Previous studies from in vitro skin models and genetically modified in vivo animal models have shown that autophagy activity was upregulated in the granular layers and contributed to terminal differentiation by degrading the organelles in the cell [24]. Disruption of autophagosome or autolysosome formation led to impaired differentiation and abnormal expression of late-differentiation markers like involucrin and loricrin [25,26].

Although UVB exposure was believed to enhance autophagy to clear damaged cellular fragments, evidence indicated that excessive UVB exposure suppressed autophagy activities [27]. Consistent with this notion, results from keratinocytes showed that 200 mJ/cm^2^ UVB exposure reduced autophagy activity (Figure 4a) and decreased the expression of *LC3* (Figure 4b), suggesting impaired autophagosome formation process. Previous studies have suggested that oxidative stress disrupted autophagic function [28] and glutathione, one of the major antioxidant defense systems in epidermis, might interact with autophagy to protect the skin barrier under oxidative stress conditions, highlighting the potential involvement of autophagy in LCC-mediated UV protection. In line with this hypothesis, LCC treatment restored *LC3* expression level, whereas co-treatment with CQ, an inhibitor of autolysosome degradation [29], attenuated this effect, suggesting that LCC can activate autophagy to protect skin against UVB exposure. It was further evaluated whether autophagy is required for LCC-mediated restoration of epidermal differentiation. LCC recovered the gene expression of *AQP3*, *IVL*, *LOR*, and *TGM1* following UVB exposure, but this rescue effect was impaired by CQ (Figure 4c–f). In fully differentiated LSE models, immunostaining further validated that LCC reversed UVB-induced reductions in transglutaminase 1, loricrin, and involucrin. However, these restoration effects were consistently compromised when autophagy was pharmacologically inhibited (Figure 5). These results collectively support the conclusion that LCC protects skin barrier function, especially keratinocyte differentiation, at least in part through restoring autophagy.

Overall, our findings demonstrate that LCC is a potent epidermal protectant under UVB stress. Beyond boosting glutathione levels and mitigating oxidative damage, LCC possesses the ability to enhance autophagy, which contributes to its protective effects against UVB-induced epidermal barrier damage, further suggesting the therapeutic potential of LCC in maintaining barrier integrity under stress conditions. However, based on our current data, several questions remain to be addressed in future studies: (1) although autophagy inhibitors interfered with LCC’s protective functions, the precise upstream mechanisms through which LCC activates autophagy require further exploration; (2) LCC restored aquaporin 3 levels in UVB-irradiated LSE even in the presence of autophagy inhibitors, suggesting additional autophagy-independent mechanisms involved in skin protection function carried out by LCC; and (3) while our study utilized pure LCC in in vitro systems, its clinical efficacy and safety as a topical skincare ingredient should be validated in clinical trials.

## 5. Conclusions

This study demonstrated that LCC activated autophagy activity and effectively prevented UVB-induced impairments in epidermal differentiation. Under UVB irradiation, LCC restored proteins involved in epidermal differentiation, hydration and proliferation. Transcriptomic and autophagy activity assays indicated that LCC enhanced autophagy in keratinocytes. Remarkably, the ability of LCC to rescue epidermal differentiation markers was abolished when autophagy was inhibited, underscoring the essential role of autophagy in the protective mechanism of LCC. Overall, LCC safeguards the skin barrier under UV stress by promoting autophagy and supporting proper keratinocyte differentiation.

## Data Availability

The data cannot be made publicly available because they contain commercially sensitive information, the data are available upon reasonable request from the authors.

## Supplementary Materials

The following supporting information can be downloaded at: https://www.mdpi.com/article/10.3390/biology15080601/s1.

## Abbreviations

The following abbreviations are used in this manuscript:

LCC	Lysine carboxymethyl cysteinate
FLG	Filaggrin
TGM1	Transglutaminase 1
AQP3	Aquaporin 3
IVL	Involucrin
CQ	Chloroquine
NT	Non-treated
